# The Dark Side of Cell Fusion

**DOI:** 10.3390/ijms17050638

**Published:** 2016-04-28

**Authors:** Daniel Bastida-Ruiz, Kylie Van Hoesen, Marie Cohen

**Affiliations:** Department of Gynecology Obstetrics, Faculty of Medicine, University of Geneva, ‎Geneva 1211, Switzerland; daniel.bastida@etu.unige.ch (D.B.-R.); kylie.vanhoesen@gmail.com (K.V.H.)

**Keywords:** cancer, cell fusion, metastasis, drug resistance, syncytin

## Abstract

Cell fusion is a physiological cellular process essential for fertilization, viral entry, muscle differentiation and placental development, among others. In this review, we will highlight the different cancer cell-cell fusions and the advantages obtained by these fusions. We will specially focus on the acquisition of metastatic features by cancer cells after fusion with bone marrow-derived cells. The mechanism by which cancer cells fuse with other cells has been poorly studied thus far, but the presence in several cancer cells of syncytin, a trophoblastic fusogen, leads us to a cancer cell fusion mechanism similar to the one used by the trophoblasts. The mechanism by which cancer cells perform the cell fusion could be an interesting target for cancer therapy.

## 1. Introduction

Cancer progresses through the accumulation of genetic alterations [1] and chromosomal instability [2] among various other factors. Tumor cells have been known for decades to have the capacity to fuse with each other and with non-tumor cells, but how the fusion of tumor cells drives the biology of cancer is not yet very clear [3]. Cell fusion has recently gained a great deal of attention for its role in cancer progression as a source of genetic instability, as well as a mechanism of metastasis and drug resistance [3,4]. Cell fusion, the topic of discussion in this review, occurs when cell membranes merge and the cytoplasm is mixed, forming multinucleated cells [5]. Cancer cells can fuse with normal cells (stromal, epithelial, macrophages), and with other cancer cells. Depending on the cell type in the fusion event, the hybrid has novel properties [6] and increased heterogeneity [7]. Cancer cells that fuse together can contribute to the formation of polyploid giant cancer cells (PGCCs), which are highly tumorigenic and chemoresistant [8]. Cancer metastasis can be initiated by the fusion of cancer cells and macrophages [4].

An immediate result of cell fusion is polyploidy. Polyploid cells are defined as having genomes with multiples of the typical diploid (2n), such as 4n or 8n [9,10]. Polyploidy is common in plants, but it is rare to find whole-body polyploidy in mammals. However, polyploidy is found in some mammalian tissue as a part of normal development and differentiation [11], including skeletal muscle, heart, placenta, liver, brain and blood cells [10]. On the other hand, polyploidy cells can form in mammals from stress, aging, and disease [12].

**Figure 1:**
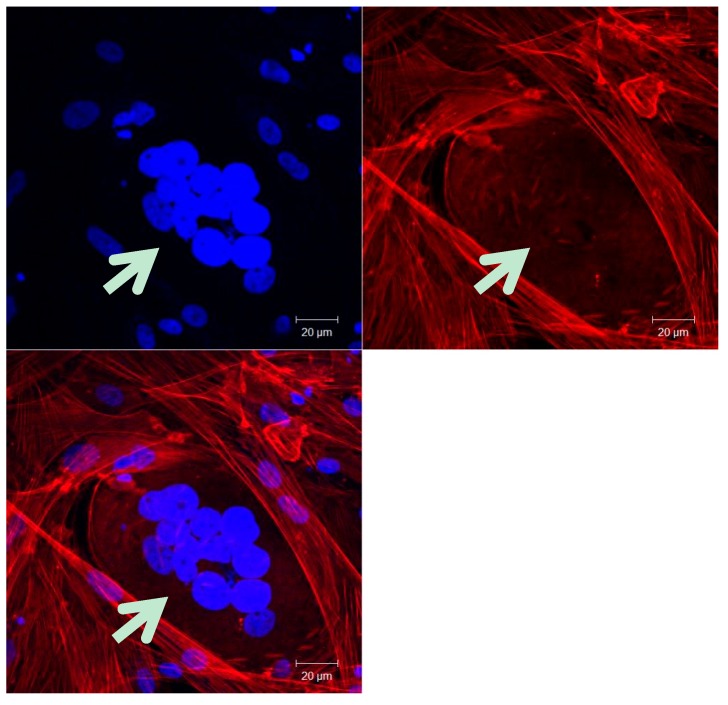
Multinucleated human ovarian cancer cell isolated from malignant ascites. The cells are labeled with DAPI (nucleus, blue) and Phalloidin (actin, red) and observed by confocal microscopy at magnification 400×. Scale bars: 20 µm. The white arrow indicates the multinucleated cell.

Polyploidy is associated with disease, such as cancer (Figure 1), because polyploid cells are genetically unstable due to the abnormal number of chromosomes. Polyploidy is an intermediate karyotype that often occurs between healthy diploid cells and neoplastic aneuploidy cells [7,10,13,14]. Aneuploidy is the presence of an abnormal chromosome number that deviates from the typical diploid number caused after the subsequent divisions of the polyploid cell, and is frequently observed in human cancer cells [12]. Cancers that have experienced a genome doubling (tetraploidy) have more chromosomal instability compared to diploid cancers, and in the majority of colorectal cancer, tetraploidization occurs as an early event [7,15].

There are four mechanisms that can lead to polyploid cells: cell fusion (as previously mentioned); endoreplication; abortive phagocytosis; and deficiencies that result in abortive cell cycles. Briefly, endoreplication occurs when DNA replication is uncoupled with cell division [12]. It is a variation of the cell cycle that generates the polyploid genome through multiple rounds of DNA replication in the absence of cellular division. One way that polyploidy is linked to cancer progression is through unscheduled endoreplication that promotes tumorigenesis by enabling chromosomal instability [9,16]. Endoreplication can either support or suppress tumorigenesis depending on the tissue environment and genetic background, specifically the presence of functioning p53 [9,17]. There are several defects that may lead to an abortive cell cycle, including mitotic slippage and cytokinesis failure [10,12]. Mitotic slippage occurs after a prolonged metaphase and the failure to satisfy the spindle assembly checkpoint. This can cause the cell to slip out of mitotic arrest and lead to a tetraploid nucleus. Cytokinesis failure can be caused from varying defects, but all result in the failure to separate the sister cells and instead form a multinucleated cell [10].

Cell fusion as a source of cancer progression is an idea that has been around since the early 1900s, though it lost attention until recently [3]. The hypothesis presenting that the development and progression of cancer predominantly reflects accumulation of random mutations and defects in DNA repair, associated with proliferation of progenitor cells [18] had stronger support in the scientific community, and now the hypothesis is being enlarged to include the contribution of cell fusion in cancer development and progression. Not only is there evidence of how cell fusion influences cancer progression, but as we will explain in this review, several features of cancer cells, especially metastasis, are better explained by cell fusion events contributing to the accumulation of random mutations. This new interest in cell fusion as part of cancer development has brought with it an increased number of studies trying to elucidate the mechanisms behind cell fusion. Several studies in animal and *in vitro* revealed that the fusion frequency in tumors was about 1% [19,20,21,22]. This data—together with the hypothesis that only 1% of the tumor hybrid cells survive, proliferate and exhibit novel properties [19]—suggests that only 0.01% of the tumor cells will be tumor hybrid cells with new properties [23]. New knowledge in the cancer field, especially concerning the tumor microenvironment, suggested that the cell fusion rate of tumor hybrid cells was likely to be higher than 0.01%. Recent results demonstrated in a xenograft assay that about 6% of the tumor cells were identified as tumor hybrid cells and under certain conditions such as chemotherapy, the cell fusion rate could be increased to 12% [24]. This review will address the different stages of cell fusion, the effects of the tumor microenvironment, as well as the recent discoveries on fusogens and the mechanism likely involved in cancer cell fusion. It should be mentioned that cell fusion is a very complicated cellular process that not only comprises cell membrane fusion, but also includes several cell rearrangements and DNA metabolism, including autophagy or nucleophagy [25], though they are beyond the scope of this article. Cancer cell fusions also resemble other types of cell fusions, including events during trophoblastic development, and the genes and proteins in trophoblasts and cancer cells have many similarities, which will be as well covered in this review.

## 2. Cancer Cell-Cell Fusion

### 2.1. Cancer Cell-Stromal Cell Fusion

Cancers are influenced by both normal and malignant cells in local and distant microenvironments [26,27,28,29,30]. Morphological differences in tumor cells and metastases can also be attributed to interaction and fusions of cancer cells [26]. This interaction of the tumor and its surrounding stroma (endothelial, macrophages, fibroblasts) can either promote or inhibit tumor progression [26,27,28,29,30,31,32].

#### 2.1.1. Novel Hybrids

Cell-cell fusion of tumor and stromal cells is a mechanism of genetic transfer that is involved in the progression of malignancy [26]. It has been shown *in vivo* that the fusion of malignant and normal cells increases malignancy in progeny in both intra- and cross-species fusions [26,32,33]. Goldenberg *et al.*, demonstrated the transfer of seven human genes from glioblastoma cells to normal hamster host cells through heterokaryon formation [26]. Heterokaryons are cells that are formed by fusion of different types of cells [34]. These progenies were malignant and expressed in both human and hamster genes and gene products. This finding implies that cell-cell fusion is a mechanism of horizontal gene transfer and that the crosstalk of cancer cells to their stromal neighbors results in the progression of malignancy [26]. Further studies demonstrate that human genes from all human chromosomes can be transduced, transcribed, and remain functional in metastatic human hamster hybrid tumors [35].

Cell fusion is a mechanism that enables genetic material to move from one cell to another and produce viable hybrid progeny. The reciprocal genetic interchange between cancer cells and their stromal microenvironment explains the heterogeneity of cancer cells [35], which allows cancer cells to acquire distinct changes to survive and adapt to microenvironments [36]. This ability for cancer tumors to evolve on a cellular level is a critical process in cancer progression, and a possible target for therapy [7].

It is also possible that cell fusion can help to inhibit cancer progression when tumor suppressor genes are present in the hybrid. Tumor suppressor genes normally act in cells to prevent tumor development by inhibiting cell proliferation. In one study, hybrids of normal cells and tumor cells were not capable of forming tumors because of genes from the normal parent that inhibited the tumor progression. There are several different types of tumor suppressor genes that can be inactivated in a variety of cancers. For example, tumor suppressor gene *P53* may be involved in 50% of cancers [37]. However, if a normal cell still has a functioning tumor suppressor gene, the cell fusion event could possibly inhibit the tumor progression.

#### 2.1.2. Metastasis

Metastasis is arguably the deadliest component of cancer. It is responsible for nearly 90% of cancer deaths [38] because the cancer cells spread from their primary site to nearby tissues as well as distant organs [4]. One hypothesis for metastasis is the epithelial to mesenchymal transition (EMT), in which epithelial cells differentiate through biochemical changes to mesenchymal cells with phenotypes of enhanced migration and invasion, as well as resistance to apoptosis [39]. Macrophages also play an influential role in metastasis in two main ways. Tumor-associated macrophages (TAM) facilitate the metastatic cascade by preparing a pre-metastatic environment, enhancing inflammation and angiogenesis, though they are not themselves neoplastic. Macrophages also influence metastasis through cell fusion events [4,40,41,42].

Metastasis is being studied as a product of bone marrow-derived cell (BMDC) fusion with malignant tumor cells, where BMDC provides its capacity of migrating and the primary tumor cell supplies its proliferative capacity [43]. Many metastatic human cancers display similar molecular and behavioral characteristics of bone marrow-derived cells, including migration capabilities, secretion of growth factors, shape change, phagocytosis, fusogenicity, and antigen expression [4]. The most studied cell-cell fusion related with metastasis is the macrophage–epithelial cancer hybrids. Macrophages have two distinct activated phenotypes. M1 macrophages, activated by pro-inflammatory molecules, help initiate tumorigenesis by forming the inflamed microenvironment [4,41], while M2 macrophages, activated by anti-inflammatory molecules, promote tumor growth, angiogenesis, phagocytosis and have the ability to fuse with tumor cells [4,44]. A possible mechanism, that we will not address deeply in this review, of cancer hybrid cells’ formation that is different from cell-cell fusion is directly linked with the phagocytosis trait of M2 macrophages. It has been suggested that macrophages, after engulfing a cell, may abort cellular digestion and result in hybrid formation [45].

After the cell-cell fusion between a BMDC and an epithelial cancer cell, the polypoid cell loses some epithelial traits such as cell-cell adhesion of E-cadherin expression, and gains mesodermal traits, mesenchymal motility mechanism, or loss of adherence, achieved by the regulation of gene expression after the cell fusion [46,47,48]. This process is known as epithelial-mesenchymal transition (EMT) and is very helpful for the first steps of metastasis because of the gain of motility by the tumor cells [49]. The lack of local adhesion also makes the cells more deformable, which helps the migration through the different membranes and tissues. An increase in protein level, normally expressed in macrophages that allow their motility, such as melanocortin 1 receptor (MC1R), β 1,6 *N*-acetylglucosaminyltransferase V (GnT-V), and secreted protein acidic and rich in cysteine (SPARC), weres observed in hybrid cells [43]. All these changes that follow the cell-cell fusion between a BMDC and an epithelial cancer cell are very helpful for metastasis, which is a complicated, multistep process characterized by the following stages: (i) Loss of adhesion to adjacent cells; (ii) Ability to traverse the basement membrane; (iii) Migration through mesodermal matrix; (iv) Intravasation into the blood or lymph circulatory system; (v) Extravasate from the vessels and (vi) Colonize distant organs or lymph nodes [50,51]. These new acquired metastatic features allow the hybrid cell to detach from the primary tumor, intravasate into the circulatory system, extravasate at a distant capillary bed and invade and proliferate in a distant organ where it can generate a new secondary tumor (for review, see [52]).

This cell fusion phenomenon was studied with the artificial fusion of human monocytes and mouse melanoma cells, and it was concluded that genomic DNA from parental monocyte/macrophage was integrated and the genes were expressed in the hybrids [53]. Afterward, it was proposed that tumor cells fused with host myeloid cells to create hybrids with migratory capabilities and the ability to occupy distant organs [4,54,55]. The fusion of macrophages (as blood-derived mesenchymal cells) and tumor epithelial in surgically joined mice demonstrated that the transcriptome identity of the progeny was similar to both the macrophage and epithelial parent cells. Additionally, there was a set of unique transcripts different from the two parents, providing evidence of how cell fusion events create metastatic cancer phenotypes [4,36].

There is also evidence of a specific macrophage marker’s, CD163, expression in both breast and colorectal cancers, which is significantly related to advanced tumor stages and lower survival [56,57]. In a follow-up study it was shown that MCF-7 cancer cells obtained CD163 (and CD45) expression through hybridization of the cancer cells and macrophages, rather than *in vitro* paracrine cellular interaction. CD163 may also prove to be a useful cancer cell fusion biomarker in clinical contexts [57]. There is not only evidence of specific macrophage markers in hybrid cells after fusion with a macrophage, but also the hybrid cells show several macrophage traits that the tumor cells did not have prior to fusion. Phagocytosis, as previously mentioned, is a behavior of M2 macrophages that has been found in many malignant tumor cells both *in vivo* and *in vitro*. It has been reported that human cancer cells can phagocytose other tumor cells, erythrocytes, leukocytes, dead cells and other extracellular particles, conferring them a cell cannibalism property exclusive of malignant tumor cells in humans. Interestingly, cathepsins D and B, which are poor prognosis factors in cancer patients, are highly expressed by macrophages to facilitate digestion of phagocytized products, demonstrating again a higher malignancy after cell fusion (for review: [52]). Tumor cells that have macrophage properties have the potential to be the deadliest cells within a tumor [4].

Metastatic tumor cells invade distant organs in a non-random manner. The primary sites of metastasis are the lungs, liver, and bones; however, the genetic mechanism for this phenomenon is still unknown [4,50,58]. Paget first suggested the “seed and soil” hypothesis [4,50,59] in which the tumor cells (seeds) have a preference for the distant tumors (soil) that they invade [59]. The only model of metastasis that addresses this hypothesis is metastasis as a macrophage disease, because EMT, TAM, and stem cells cannot explain this pattern of metastasis [4,60]. Macrophage presence is higher in liver and lung tissues because of the higher degree of bacterial exposure and the greater damage to resident macrophages [4,61]. This is a possible explanation for these sites as preferential “soil” for metastatic cancers [4]. Another example is the fusion of myeloma cells with B lymphocytes that results in metastasis in the spleen and liver [3,62]. Additionally, cell fusion with resident cells of the distant organ allow the survival of disseminated tumor cells in new microenvironments, which go on to progress metastases when additional oncogenes are activated [3,63]. An inflamed microenvironment is a contributor to tumor cell-macrophage cell fusions, as is radiation therapy [4,64]. The decreased survival of some irradiated cancer patients is due to the enhanced cell fusion of macrophage and epithelial cells, and specifically, it has been indicated that the human brain should never be irradiated [60,65].

### 2.2. Cancer Cell-Cancer Cell Fusion

Cancer cells are highly fusogenic and can merge with other cancer cells, not limited to certain tumor types. Cancer cell fusion allows cells to quickly obtain genomic material, and changes cell genomes on a much larger scale than mutations [66]. The fusion of cancer cells can produce new acquisitions of drug resistance, multiple metastasis organotropism and clonal expansions.

Cell fusion events can contribute to cancer populations acquiring drug resistance. Some cells have the ability to resist certain drugs by activation of enzymes which can metabolize the administrated drug, by increased expression of multidrug transporters, or by lacking the drug receptor. Different cell lines that fuse can acquire the same resistance as the parents, and even the emergence of new drug resistance. For example, the *in vitro* fusion of 5-fluorouracil-resistant cells with methotrexate-resistant cells of the mammary tumor cell lines produced hybrids resistant to both drugs, creating a double-resistant cancer cell, in which elimination will be much more difficult. Interestingly, the fused cell resulting from this cancer cell-cancer cell fusion event is resistant as well to a drug that both parental cells were sensitive to, melphalan [3,19]. A more recent study showed that *in vitro* co-cultivation of hygromycin-resistant murine mammary carcinoma cells with puromycin-resistant murine BMDCs resulted in dual-resistant cells. In addition, both parental markers were found in these cells, suggesting a possible cell fusion origin, even if horizontal gene transfer cannot be rejected as a dual resistant formation hypothesis. Interestingly, the dual resistant hybrid cell showed upregulation of Abcb1a and Abcb1b multidrug transporters which conferred the cell a marked resistance towards some chemotherapeutic drugs as 17-DMAG, doxorubivin, etoposide and paclitaxel [67]. These acquisitions of new drug resistances could occur at low frequency but allows the escape of rare hybrid cells. This advantage for these hybrid cells is a source of tumor relapse [3].

In regards to the previous discussion about metastasis, it is suggested that tumor cells have the capability to occupy specific distant organs, known as metastasis organotropism [68]. In heterogeneous tumor cell populations, fusion between cancer cells can create multiple metastasis organotropisms if cells fuse with differing organ specificities. A recent study indicates that the fusion between bone-tropic and lung-tropic subpopulations results in stable hybrids with dual tropisms for both bone and lung organs [68], which express metastasis gene signatures. Multiple metastasis organotropism allows hybrids to colonize several different organs when in circulation and also to quickly re-colonize organs that have responded positively to therapies [3]. This displays another form of cell fusion, between only cancer cells, that increases metastasis and therefore the overall progression of cancer.

### 2.3. Cancer Stem Cells

Cancer stem cells (CSCs) are a rare population of cells in the tumor mass which possess the ability to initiate the growth of a heterogeneous tumor [69]. The origin of CSC remains elusive, but two potential mechanisms are described. The first one postulates that adult stem cells can accumulate genetic aberrations which will generate the CSC [70,71], and the second potential mechanism of CSC generation is through fusion between stem cells and differentiated cells [3,72,73,74]. The origin and rates of appearance of CSC are likely to be cancer specific, showing, for example, high frequency in melanoma, with a 25% of unselected patient-derived melanoma cells that could form xenograph tumors [75].

CSCs have the ability to initiate tumors, and they also present resistance to chemotherapy, thereby suggesting they may cause cancer relapse [76]. The characteristics conferring CSCs’ resistance to chemotherapy includes the slow proliferation rate, the more efficient DNA repair mechanism, the high expression of anti-apoptotic proteins and the high-level expression of ABC multidrug pumps [76]. CSCs are also thought to have some deregulated signaling pathways that control self-renewal of normal somatic cells (such as Notch-signaling [77] and Wnt/β-catenin signaling pathways [78]) resulting in uncontrolled self-renewal capacity of CSCs.

The origin of CSCs, as it was mentioned before, could be the cell fusion between a stem cell and a differentiated cell. Several options are observed: (i) a bone marrow stem cell and local differentiated cell; (ii) a bone marrow cell with a local stem cell; (iii) both local fusion partners with one of them being a stem cell; and (iv) a disseminated cell from a different tissue origin and a local stem cell. There is another possible scenario where a mutated cell with stem cell-like properties fuses with a differentiated cell. In all of the cases, one of the cells presents the important self-renewal capacity that the CSCs inherits [73].

CSCs are gaining interest because of their characteristics that not only confer them the ability of causing cancer relapse, but they are also hypothesized to be one of the causes of metastasis. Because of these different properties of CSCs, subpopulations of CSCs have been proposed. Primary CSCs (pCSCs) induce primary tumor formation and metastatic CSCs (mCSCs) induce metastases [79].

Another possible way of generating CSCs from rare cell fusion events is through polyploid giant cancer cells (PGCCs). PGCCs are large cancer cells containing multiple copies of DNA which have cancer stem-like properties [80] and are thought to generate cancer stem-like cells, contributing to cancer progression [8]. PGCCs are positive for both normal and cancer stem cell markers and have characteristics of both. For example, single PGCCs can form spheroids and generate tumors, similar to the ability of cancer stem cells [80]. Also, PGCC formation increases under multiple stressors such as antimitotic drugs, radiation, and hypoxia [80]. It is proposed that PGCCs form through cell fusion 10%−20% of the time and they can be three to ten times larger than normal cells. These giant cancer cells play a role in the cancer cell cycle and are highly tumorigenic and chemo-resistant [8].

## 3. Mechanisms of Cell Fusion

Cell fusion is one of the mechanisms that can lead to polyploidy, and, hence, cancer [1,2,3,4,9,10]. Nevertheless, polyploidy is not always a synonym of harmful consequences for the cell; on the contrary, cell fusion occurs at different levels in the organism, being essential processes for correct conception, birth and development [11].

Fecundation is the first essential cell-cell fusion event that takes places in sexual reproductive organisms. The oocyte and the spermatozoon fuse in order to mix their cellular content and generate a new living organism [81,82]. In order to develop a functional organism, several cell-cell fusion events take place [83]. In animals presenting musculature and in animals presenting skeletal system, the muscle differentiation, bone maintenance, repair and remodeling are all episodes which need the participation of cell-cell fusions [84,85,86,87]. Muscular fibers are generated by fusion of several myoblasts to form a polynucleated cell of syncytium [84,85], while osteoclasts that are in charge of bone turnover are composed of several fused macrophages [86,87]. All these cell-cell fusion events, however, do not lead to cancer and it would be very interesting to find out the reason why polyploidy in these cases does not have an oncological fate and how the cell controls the non-tumorigenic destiny of these fused cells.

### 3.1. Proteins Associated with Cell-Cell Fusion

Cell-cell fusion events are well controlled by different proteins expressed in the fusing cells. For example, in mammalian fecundation, oocyte tetraspanins CD9 and CD81 are important for the fusing event [88,89,90,91,92,93], as well as IZUMO and A disintegrin and metalloproteinase (ADAM) sperm proteins [94,95,96]. For the moment, only CD9 and IZUMO have been identified as essential proteins for sperm-egg fusion, but there is no evidence indicating that these two proteins directly interact during sperm-egg fusion [5]. In myoblasts, the Ig-class of cell adhesion molecules are essential for cell-cell recognition and fusion [97,98] and in osteoclasts, CD47, CD200, dendritic cell-specific transmembrane protein (DC-STAMP) and osteoclast-stimulatory transmembrane protein (OC-STAMP) seem to be important for the macrophage fusion [99,100,101,102,103]. DC-STAMP and OC-STAMP are the only identified transmembrane proteins essential for pre-osteoclast fusions and even though they are related, they are not interchangeable and the deficiency of one of them cannot be complemented by overexpression of the other [104,105].

In addition to the tissues generated by cell-cell fusion previously mentioned, another essential tissue that mammals need in the cell-cell fusion process is the placenta [106]. Placenta development is divided in two differentiated phases. In the first stage, several cytotrophoblasts fuse together to generate polynucleated cells called syncytiotrophoblasts. These newly generated cells are highly invasive and they enter the maternal uterus. Once the syncytiotrophoblast has invaded the maternal tissue, it becomes the outer surface of the placenta, and its role changes completely. Its location confers it the ability of exchanging oxygen, nutrients and waste products between the embryo and the mother’s vascular system, as well as the production of hormones and immune tolerance. During the performance of this second role, cytotrophoblastic cells fuse with the syncytiotrophoblast in order to renew the tissue [107,108,109,110,111]. During the first phase of placenta development, it was mentioned that the syncytiotrophoblastic cells are invasive cells that migrate and invade the maternal uterine decidua [107,108,109]; curiously, migration and invasion are also features that the cancer cells are able to perform [3,4,112]. Furthermore, the cell-cell fusion of cytotrophoblasts has been deeply studied and in contrast with the previously mentioned cell-cell events, essential bona fide fusogens have been found in the syncytiotrophoblast fusion formation event [113,114]. These fusogens are class I envelope (env) human endogenous retroviruses (HERV) that were acquired in humans from retroviruses [113,114,115] and act similarly to target-soluble *N*-ethylmaleimide-sensitive factor attachment protein receptor (t-SNARE) and vesicular-SNARE (v-SNARE) [116]. These two proteins mediate the fusion of intracellular membranes by forming bundles of alpha-helixes, which result in membrane apposition and fusion [116,117]. Interestingly, engineered flipping of t-SNARE and v-SNARE into the surface of cell membranes has been shown to promote cell-cell fusion [116,118,119]. Two identified cell-cell fusogens that appear to use a similar α-helixes fusion mechanism are syncytin 1 (acquired 19−28 million years ago) [113,114,120] and syncytin 2 (acquired 40 million years ago) [115] and are part of the 8% of the human genome that were acquired from retroviruses [121]. Interestingly, in addition to the similar features between syncytiotrophoblast and cancer cells (migration and invasion), several studies have found a high expression of syncytin 1, syncytin 2 and their receptors in different cancers, supporting an important function of this envelope human endogenous retrovirus (env HERV) proteins in cancer progression [122,123,124,125,126,127,128,129,130,131] (Table 1). Different human breast cancer cell lines and 38% of the breast cancer specimens studied expressed syncytin [129]. The alanine, serine and cysteine selective transporter 2 (ASCT2) receptor of syncytin 1 was furthermore expressed in endothelial and cancer cells [130,132,133,134,135], allowing a demonstrated fusion between breast cancer cells and endothelial cells that can be inhibited by syncytin inhibitory peptides [130]. Additionally, in endometrial carcinoma tissues, syncytin 1 is upregulated in both malignant and benign tumors, as well as ASCT1 and ASCT2 expression. But these are not the only types of cancers that have been reported to overexpress envelope HERV proteins or have their receptors in cells where basal expression is lower. There is even expression of envelope HERV protein in cell types where its expression is normally absent. The different cell lines that were found to express syncytin 1 are summarized in Table 1; even though they have not been demonstrated to fuse in the different cancer tissues, they probably have the ability to fuse with other cells expressing syncytin 1 receptors. The reason why these envelope HERV proteins are overexpressed or expressed is not completely clear, but a hypothesis suggests that the abnormal number of chromosomes or mutations in cancer cells change the protein expression pattern, affecting the expression of these envelope HERV proteins and their receptors [135].

Moreover, other proteins have been identified as playing an important role in the syncytialization process. E-Cadherin, cadherin-11, zona ocludens-1 (ZO-1), connexin-43 and 78 kDa glucose-regulated protein (GRP78) are some of these proteins involved in the cell-cell fusion process [107,136,137,138]. GRP78, a reticulum endoplasmic protein, has been found in the surface of trophoblastic cells where it plays a role in cell fusion. Interestingly, cancer cells also express this protein at their surface, probably displaying a function in cancer cell fusion [136].

### 3.2. Syncytin-Dependent Fusion Mechanism

Syncytin 1 (HERV-W) and syncytin 2 (HERV-FDR) are envelope human endogenous retrovirus proteins characterized by binding to the receptor and achieving the membrane fusion in a pH-independent manner [106,107]. The sophisticated mechanism that class I envelope proteins use to fuse the membranes is well defined in viruses, and the high homology of the envelope HERV with the retroviral envelope proteins implies the use of the same or very similar mechanism of fusion [106,107,139,140]. Cell fusion in cancer is poorly understood, but certain steps and cellular components can be surmised from the fusion events already understood.

**Figure 2:**
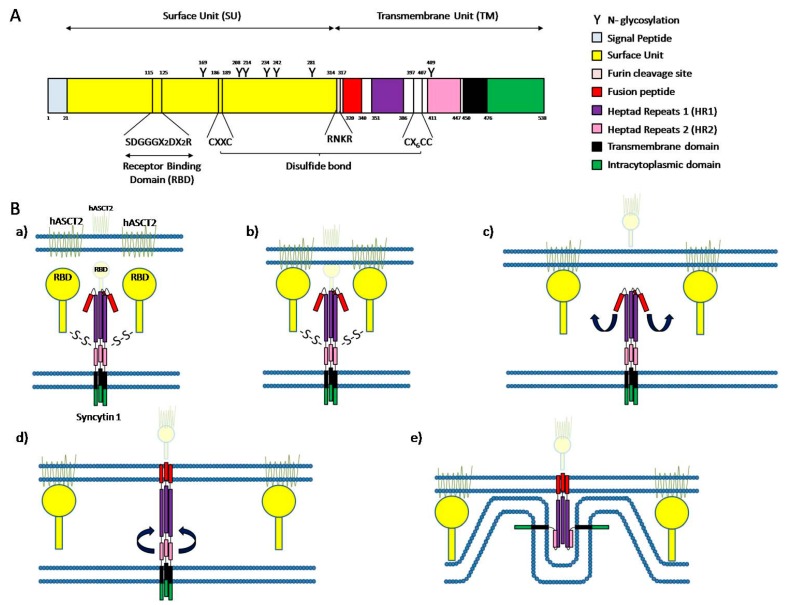
Cell fusion mediated by syncytin 1. (**A**) Schematic portrait of syncytin 1 structure. The signal peptide domain is represented in light blue. The surface unit (SU) is represented in yellow and contains a receptor binding domain (RBD; SDGGGX2DX2R) and a CXXC motif. The transmembrane unit (TM) contains a fusion peptide represented in red, heptad repeats 1 (HR1) represented in purple, heptad repeats 2 (HR2) represented in pink, a transmembrane domain represented in black, an intracytoplasmic domain represented in green and a CX6CC domain. Between the SU and the TM is a furin cleavage site (RNKR) represented in light red. The Y indicate *N*-glycosylation sites and numbers indicate the amino acid position. A disulfide bond is formed between the CXXC motif of SU and the CX6CC domain of TM; (**B**) Schematic representation of syncytin 1-dependent cell fusion. (**a**) Resting stage; (**b**) RBD (yellow) binding to the hASCT2 receptor (light green); (**c**) Disulfide bond breaking and removing of SU domains, producing a conformational change in syncytin 1 protein leading to the insertion of the fusion peptide (red) into the cell membrane; (**d**) Assembly of HR2 (pink) and HR1 (purple); (**e**) Final stage with the membranes in close proximity and initiation of membrane bending. Adapted from [106,107].

Syncytin-1-dependent fusion mechanism has been better characterized than syncytin-2-dependent fusion mechanism, so we will focus on the first one. Syncytin-1, like all the retroviral envelope proteins, is a glycoprotein composed of a surface unit (SU) and a transmembrane unit (TM). The SU contains a receptor binding domain (RBD), a furin cleavage site (^314−^RNKR^−317^), 6 *N*-glycosylation sites and a C_ΦΦ_C (^186−^CX_2_C^−189^) motif. On the other hand, TM contains a fusion peptide (FP), two heptad repeats (HR1 and HR2), a transmembrane anchorage domain (tm), a cytosolic tail (cyt), 1 *N*-glycosylation site and a ^397−^Cx_6_C^−407^ domain. SU and TM are translated together, but SU contains a furin cleavage site, a conserved cleavage site that can be cleaved by proteases like furin-convertase enzyme separating both units (Figure 2A). The protein is translated and folded in the endoplasmic reticulum, where the cleavage of both subunits is done prior to a disulfide bond formation between the ^397−^Cx_6_C^−407^ domain of TM and ^186−^CX_2_C^−189^ motif of SU [106,107,139,140]. Functional syncytin 1 is a trimer, in which trimerization takes place in the endoplasmic reticulum. After maturation in Golgi apparatus, the mature functional protein is transported to the membrane where it can trigger cell-cell fusion [106].

In order for cell-cell fusion to take place, syncytin 1 has to recognize a receptor in the target membrane, which in the case of this protein can be either ASCT1 or ASCT2. The receptor binding domain located in the SU subunit of syncytin 1 recognizes its particular receptor located in the target membrane and a conformational change in the trimer produces the dissociation of the SU subunit and the TM subunit by breakage of the disulfide bond. This structure modification generates a loop-to-helix movement of the syncytin 1 fusion peptide causing its projection toward the top of the glycoprotein, interacting with the target membrane and strongly inserting into it [106,107]. Once both membranes are firmly connected through the envelope protein, the HR2 domain folds back and interacts with the HR1 domain, reversing the direction of the cell membrane and bringing both membranes in close proximity [106] (Figure 2B). The proximity of the membranes considerably reduces the free energy needed to overcome the barrier to merge [106,107].

### 3.3. Model of Membrane Fusion

The first membrane-merging step consists of a hemifusion where only the outer leaflets of both membranes are fused. The close proximity of the membranes and the contact of the opposing outer cell membranes causes dehydration of the contact site, reducing the hydration repulsion between the outer leaflets of the membranes. This reduction of repulsion leads to the formation of a fusion stalk, where only the outer leaflets of the membranes are fused [106,107,141]. Nevertheless, the close proximity of cell membranes and dehydration are not enough for the hemifusion to take place. The localization of negatively charged phospholipids as phosphatidylserine in the outer membrane has been shown to be required for syncytial fusion as well as for skeletal muscle formation and, interestingly, trophoblast-derived BeWo choriocarcinoma cell line fusion. This redistribution of phosphatidylserine from its normal location in the inner leaflet to the abnormal location in the outer membrane is called phosphatidylserine flip [106,107,141,142,143,144]. After the formation of a fusion stalk facilitated by all of these different events, radial expansion of the fusion stalk occurs, and it ends up in the appearance of a hemifusion diagram where the inner leaflets are still separated. Finally, the inner leaflets fuse as well, causing a complete merge of the membranes and the opening of a pore by which the cell content can mix, finally achieving the cell-cell fusion [106].

## 4. Discussion

Cell-cell fusion is a necessary activity which needs to be controlled in order to perform correctly, or medical conditions may result. The importance of cell-cell fusion spans from viral survival, to fecundation of gametes, to the correct development of the embryo and the adult [81,82,83,84,85,86,87]. Wrong cell-cell fusion in trophoblast can generate difficulties in the pregnancy, both for the embryo and the mother, such as preeclampsia [145,146,147]. While wrong cell-cell fusion of osteoclasts generates bone turnover problems, including osteoporosis [148], wrong cell-cell myoblast fusion generates malformation of the muscles [84,85]. All of these events are very complicated and their control should be strictly supervised.

Recent studies have revealed some of the effectors that are implicated in the control of cell-cell fusion, but the spotlight is centered on uncovering the proteins implicated in the direct fusion: the fusogens. The investigations are going in the right direction with new effectors appearing continuously, but the identification of bonafide fusogens is still not achieved. Great advances have been made in the identification of fusogens and adjuvant proteins implicated in cell-cell fusion during fecundation, with CD9 and IZUMO as promising proteins [88,89,90,91,96]. In other cell-cell fusion, such as those in muscle fibers or bone, identification is taking longer, even with the understanding of these cell-cell fusions [97,98,99,100,101,102,103]. The only bonafide fusogen that has been found is the one implicated in the syncytiotrophoblast fusion, syncytin 1. This envelope HERV protein has been found to drive the direct interaction between membranes, bringing them together and making them fuse [113,114,120].

It is interesting how this envelope HERV protein, together with other env HERV proteins in the human genome, can generate the fusion of cells for something as important as placenta formation, and how, at the same time, it can be used by other cells to generate cancer or metastasis [125,127,128,129,130,131].

The lack of control in the expression of env HERV proteins and their receptors can lead to cell-cell fusion that will generate aneuploidy cells that will in the end generate cancer tumor cells. It is surprising how an uncontrolled expression of a few proteins that are necessary for correct development, can be used by cancer cells to induce tumor development and metastasis [125,127,128,129,130,131,132,133,134,135].

Cell-cell fusion is not only implicated in the generation of cancer cells but also in the progression of cancer. Cancer cell fusion with other cells in the body, such as BMDC, can lead to metastasis and increased malignancy [43,49,51]. The cell fusion of tumorigenic cells and BMDC leads to metastasis through the combination of the activities of each of the mixing cells. The tumorigenic cell supplies the proliferation activity while the BMDC supplies the migration and invasion activity [43]. These cell-cell fusions are very convenient for the cancer tumors as they are able to assure their survival in the body by colonizing new organs [4,44].

Even though virus-cell fusion mechanisms are already well defined, and cell-cell fusion mechanisms are only partially understood, very little about fusion mechanisms in cancer cells has been described. Nevertheless, all the different proteins implicated both in cancer cell fusion and in physiological cell fusion leads us to recognize high resemblance between the fusion mechanisms used by physiological cells, especially syncytiotrophoblast, and cancer cells [125,127,128,129,130,131]. For the moment, investigations trying to elude the cancer cell fusion mechanisms are not very advanced, but the alikeness of the two processes permit us to use the placenta trophoblast fusion as a basis for cancer cell fusion investigation. The study of the cancer cell fusion mechanisms could be interesting for a better general understanding of cancer, as well as a potential treatment against the disease.

Although surgery and chemotherapy treatments against cancer cells are reducing the mortality of this disease, we are still far from having a thorough understanding of the disease and finding a way to eradicate it. Chemotherapy treatments against cancer cells are very useful in reducing the tumor size or completely eliminating it by attacking the cancer cells directly [149]. However, resistance to drugs is a feature that some of the cancer cells have and, at least *in vitro*, probably through cell fusion, double-resistant cells can be generated leading to a more complicated cell to eliminate [3,19,67].

Until now we have commented on how cell-cell fusion has turned against us by generating hybrid cells that can lead to one of the most lethal diseases, from something that is completely necessary for our development. Recent studies are trying to elucidate a way to turn cell-cell fusion to our benefit by counterattacking the cancer cells with artificially created fused cells [150,151].

The cell-cell fusion events happening in our body to generate cancer cells cannot be predicted and thus, we cannot anticipate stopping them from happening. But we can attack them once the fused cancer cells have already been originated. Dendritic cells (DC) are the most potent antigen-presenting cells and are able to initiate primary immune response [152,153]. If tumor cell antigens are loaded into DC, these DC will induce antitumor immunity and induce tumor regression after recognition of the antibodies expressed by tumor cells [150]. In order to have DC expressing the tumor cell antigens, a cell-cell fusion strategy can be used. By fusing these two types of cells with polyethylene glycol [151,154], electrofusion [155,156,157] or virus-mediated fusion [158,159], DC will process and present the tumor antigens, allowing the body to be prepared to recognize and attack the tumor cells [150,151,154,155,156,157,158,159].

There are still a lot of questions to answer about how cell-cell fusion is induced to generate polyploid cancer cells, how it takes place and, especially, how to stop it. Although the results recently obtained are promising and help us better understand the process, there is still a long way to go before completely understanding it, especially since cell-cell fusion occurs at all tissue levels and all cell types, so no generalities can be concluded. Regardless, little by little, more questions will find their answers and the puzzle will be finally solved.

## Figures and Tables

**Table 1:** Expression of syncytin in different human cancers.

Protein	Human Tissue or Cell Line	References
Syncytin 1	Cutaneous T-cell lymphoma	[122]
Syncytin 1	Urothelial cell carcinoma	[123]
Syncytin 1	Endometrial carcinoma	[124,125]
Syncytin 1	Leukemia cells	[126,127]
Syncytin 1	Lymphoma cells	[126,127]
Syncytin 1	Colorectal carcinoma	[128]
Syncytin 1	Breast cancer	[127,129,130]
Syncytin 1	Ovarian carcinoma cell line	[127,131]
Syncytin 1	Gastric cell line	[127,131]
Syncytin 1	Lung carcinoma cell line	[127,131]
Syncytin 1	Brain carcinoma cell line	[127]
	Cervix carcinoma cell line	
	Melanoma cell line	
	Prostate cancer cell line	
	Pancreatic carcinoma cell line	
	Hepatocellular carcinoma cell line	
	Kidney carcinoma cell line	
	Squamous carcinoma cell line	
	Colon carcinoma cell line	
	Bladder carcinoma cell line	
Syncytin 2	Endometrial carcinoma	[124]

## Abbreviations

Abcb1a	ATP-binding cassette b1a multidrug transporter.
Abcb1b	ATP-binding cassette b1b multidrug transporter.
ADAM	A disintegrin and metalloproteinase
ASCT1	Alanine, Serine and Cysteine selective transporter 1
ASCT2	Alanine, serine and cysteine selective transporter 2
BMDCs	Bone marrow-derived cells
CD	Cluster of Differentiation
CSCs	Cancer stem cells
Cyt	Cytosolic tail
DC	Dendritic cells
DC-STAMP	Dendritic cell-specific transmembrane protein
DNA	Deoxyribonucleic acid
EMT	Epithelial to mesenchymal transition
Env	Envelope
FP	Fusion peptide
GnT*-*V	β 1,6 *N*-acetylglucosaminyltransferase V
GRP78	78 kDa glucose-regulated protein
HERV	Human endogenous retrovirus
HR1	Heptad repeats
HR2	Heptad repeats
LD	Linear dichroism
MC1R	Melanocortin 1 receptor
mCSCs	metastatic cancer stem cells
OC-STAMP	Osteoclast-stimulatory transmembrane protein
pCSCs	Primary cancer stem cells
PGCCs	Polyploid giant cancer cells
RBD	Receptor binding domain
SPARC	Secreted protein acidic and rich in cysteine
SU	Surface unit
t-SNARE	target-soluble *N*-ethylmaleimide-sensitive factor attachment protein receptor
TAM	Tumor-associated macrophages
TM	Transmembrane unit
Tm	Transmembrane anchorage domain
v-SNARE	vesicular-soluble *N*-ethylmaleimide-sensitive factor attachment protein receptor
ZO-1	Zona ocludens-1
